# Malignant phyllodes tumors display mesenchymal stem cell features and aldehyde dehydrogenase/disialoganglioside identify their tumor stem cells

**DOI:** 10.1186/bcr3631

**Published:** 2014-03-26

**Authors:** Jin-Jin Lin, Chiun-Sheng Huang, John Yu, Guo-Shiou Liao, Huang-Chun Lien, Jung-Tung Hung, Ruey-Jen Lin, Fen-Pi Chou, Kun-Tu Yeh, Alice L Yu

**Affiliations:** 1 Genomics Research Center, Academia Sinica, 128 Academia Road, Section 2, Taipei 11529, Taiwan; 2 Institute of Biochemistry and Biotechnology, College of Medicine, Chung Shan Medical University, Taichung, Taiwan; 3 Department of Surgery, College of Medicine, National Taiwan University, Taipei, Taiwan; 4 Department of Surgery, Division of General Surgery, Tri-Service General Hospital, Taipei, Taiwan; 5 Department of Pathology, College of Medicine, National Taiwan University, Taipei, Taiwan; 6 Institute of Stem Cell & Translational Cancer Research, Chang Gung Memorial Hospital at Linkou, Taoyuan, Taiwan; 7 Department of Surgical Pathology, Changhua Christian Hospital, Changhua 135, Nansiao St, Changhua 500, Taiwan

## Abstract

**Introduction:**

Although breast phyllodes tumors are rare, there is no effective therapy other than surgery. Little is known about their tumor biology. A malignant phyllodes tumor contains heterologous stromal elements, and can transform into rhabdomyosarcoma, liposarcoma and osteosarcoma. These versatile properties prompted us to explore their possible relationship to mesenchymal stem cells (MSCs) and to search for the presence of cancer stem cells (CSCs) in phyllodes tumors.

**Methods:**

Paraffin sections of malignant phyllodes tumors were examined for various markers by immunohistochemical staining. Xenografts of human primary phyllodes tumors were established by injecting freshly isolated tumor cells into the mammary fat pad of non-obese diabetic-severe combined immunodeficient (NOD-SCID) mice. To search for CSCs, xenografted tumor cells were sorted into various subpopulations by flow cytometry and examined for their *in vitro* mammosphere forming capacity, *in vivo* tumorigenicity in NOD-SCID mice and their ability to undergo differentiation.

**Results:**

Immunohistochemical analysis revealed the expression of the following 10 markers: CD44, CD29, CD106, CD166, CD105, CD90, disialoganglioside (GD2), CD117, Aldehyde dehydrogenase 1 (ALDH), and Oct-4, and 7 clinically relevant markers (CD10, CD34, p53, p63, Ki-67, Bcl-2, vimentin, and Globo H) in all 51 malignant phyllodes tumors examined, albeit to different extents. Four xenografts were successfully established from human primary phyllodes tumors. *In vitro*, ALDH^+^ cells sorted from xenografts displayed approximately 10-fold greater mammosphere-forming capacity than ALDH^-^ cells. GD2^+^ cells showed a 3.9-fold greater capacity than GD2^-^ cells. ALDH^+^/GD2^+^cells displayed 12.8-fold greater mammosphere forming ability than ALDH^-^/GD2^-^ cells. *In vivo*, the tumor-initiating frequency of ALDH^+^/GD2^+^ cells were up to 33-fold higher than that of ALDH^+^ cells, with as few as 50 ALDH^+^/GD2^+^ cells being sufficient for engraftment. Moreover, we provided the first evidence for the induction of ALDH^+^/GD2^+^ cells to differentiate into neural cells of various lineages, along with the observation of neural differentiation in clinical specimens and xenografts of malignant phyllodes tumors. ALDH^+^ or ALDH^+^/GD2^+^ cells could also be induced to differentiate into adipocytes, osteocytes or chondrocytes.

**Conclusions:**

Our findings revealed that malignant phyllodes tumors possessed many characteristics of MSC, and their CSCs were enriched in ALDH^+^ and ALDH^+^/GD2^+^ subpopulations.

## Introduction

Breast phyllodes tumors (PTs) are rare neoplasms [1], representing less than 1% of all primary breast tumors in western countries [2]. However, an incidence rate of 6.92% was reported in a Singaporean study, suggesting its higher frequency among Asian women [3]. The World Health organization classified breast PTs into benign, borderline and malignant histopathologically [4]. However, there are occasional discrepancies between the clinical behavior and histopathological parameters of PTs, and the progression rate and outcomes of PTs remain unpredictable [1]. So far, there is no effective therapy other than surgery [5]. While all grades of breast PTs have the potential for local recurrence, only borderline and malignant PTs were shown to metastasize to other organs, such as lungs, bone and liver [6]. The metastatic PTs may show a resemblance to osteogenic sarcoma, chondrosarcoma, liposarcoma, leiomyosarcoma or rhabdomyosarcoma [7], which is attributed to the inherent heterogeneity within the primary PTs [1]. However, there has been no report of neural differentiation of malignant PTs. The versatile property of PTs to convert into various sarcoma types is reminiscent of the features of mesenchymal stem cells (MSCs). It has been well-documented that MSCs may differentiate into adipocytes, osteocytes and chondrocytes [8]. Subsequent studies demonstrated that MSCs can even be induced to neuron-like cells differentiation [9]. This led us to hypothesize that malignant PTs may possess MSC-like properties. Recently, GD2, a disialoganglioside has been identified as a marker for stem cells of MSCs [10] and breast cancer [11]. It will be of interest to determine whether GD2 is expressed in PTs and their stem cells.

Cancer stem cells (CSCs) have the capacity to create bulk tumors through self-renewal and differentiation [12]. A successful cancer therapy must thus eliminate these cells. The identification and isolation of CSCs thus become important in the treatment of malignant PTs. Although several markers have been successfully used to enrich cancer stem cells from various cancers, CSC markers for PTs have yet to be deciphered. In this study, we investigated the expression of a variety of markers in malignant PTs and searched for CSC markers for PTs.

## Methods

### Clinical specimens of malignant PT

All human breast cancer specimens were obtained from patients with malignant PT who had undergone initial surgery at the Tri-Service General Hospital (Taipei, Taiwan), National Taiwan University Hospital (Taipei, Taiwan), Chunghua Christian Hospital (Chunghua, Taiwan). Samples were fully encoded to protect patient confidentiality and were utilized under a protocol approved by the Institutional Review Board of Human Subjects Research Ethics Committees of Academia Sinica (Taipei, Taiwan) and collaborating medical centers. We have confirmed that informed written consent was obtained from those patients who provided fresh tumor specimens and that the IRB exempted the informed consent from patients who provided paraffin-embedded tissue sections.

### Animal model

Female NOD-SCID (non-obese diabetic-severe combined immunodeficiency; Tzu Chi University, Hualien, Taiwan mice were purchased from Jackson Lab, Bar Harbor, ME, USA) and housed under specific pathogen-free conditions in the Animal Center of the Institute of Cellular and Organismic Biology of Sinica. We developed an orthotropic xenograft model as described by Kuperwasser *et al.*[13]. Briefly, fat pads were cleared and injected with a mixture of human primary cancer cells, human mammary stromal cells and Matrigel (BD 356237, 2.5mg/ml, USA)®. The human mammary stromal cells were obtained from patient BC515 who had undergone initial surgery. The tumor specimens were sliced to square (1 mm^2^) then subjected to enzymatic digestion by being incubated in RPMI1640 medium containing collagenase (Sigma C5138, 1,000 U/ml, USA), hyaluronidase (Sigma H3884, 300 U/ml), and DNase I (Sigma DN25, 100 μg/ml) at 37°C for one hour. After filtration through a 100-μm cell strainer (BD Biosciences, USA), primary breast tumor cells were collected and resuspended in RPMI1640 medium supplemented with 5% FBS, and then injected into mammary fat pads of NOD-SCID mice. The animals were monitored weekly for tumor growth. Tumor cells from the xenografted mice were harvested in a similar manner and injected into other mice for serial passages. Mice were treated in accordance with the Institutional Animal Care and Use Committee of the Academia Sinica guidelines for experiments and approved by a committee of the same office.

### Immunohistochemical analysis

Immunohistochemical analysis was performed on formalin-fixed paraffin-embedded tissue. Sections (3 μm) on coated slides were deparaffinized and rehydrated then subjected to antigen retrieval by autoclave or microwave in alkaline buffer pH9 (antigen Retrieval AR10, BioGenex, Fremont, CA, USA) for 10 minutes. After antigen retrieval, sections were treated with H_2_O_2_ to block the endogenous peroxidase activity. After washing out the H_2_O_2_, the sections were incubated with diluted primary antibodies as indicated by the manufacturer at room temperature for one hour, followed by staining with Super Sensitive Polymer-HRP Detection System (BioGenex), counter-staining with Mayer’s hematoxylin and mounted in glycerin. The primary antibodies used included the following: CD44 (DF1485, DAKO, USA), CD29 (O.N.98, US Biological), CD106 (3H1814, US Biological), CD166 (MOG/07, Novocastra, USA) CD105 (SN6h, DAKO), CD90 (3F102, US Biological), GD2 (14G2a, Bio Technetics, San Diego, CA 92121), ALDH1 (44/ALDH, it recognizes all ALDH1 isoforms, BD, USA), Oct-4 (240408, Santa Cruz, USA), CD117 (polyclonal c-Kit, DAKO), CD10 (56C6, BioCarta, USA), P53 (DO-7, DAKO), P63 (4A4, DAKO), Ki-67 (MIB-1, DAKO), bcl-2 Oncoprotein (124, DAKO), Globo-H (MBr1, ALEXIS, USA), CD34 (QBEnd 10, DAKO), vimentin (Vim3B4, DAKO), collagen type II (polyclonal: 1 fibrillar collagen NC1 and 1VWFC, Abcam, UK), nestin (196908, R&D and polyclonal, Santa Cruz Biotechnology, USA), βIII-tubulin (Tuj-1, R&D and polyclonal, Millipore, USA) and glial fibrillary acidic protein (GFAP) (273807, R&D and polyclonal, Millipore). Sections were examined by pathologists.

### Immunofluorescent staining

Cells were fixed in 4% paraformaldehyde at room temperature for 10 minutes. Primary antibodies were used at the dilutions suggested by the manufacturer. Cells were permeabilized with a permeabilization buffer (eBioscience) before staining with ALDH1, collagen type II, nestin, βIII-tubulin and GFAP. Secondary antibodies (1:100) labeled with Alisa488, Alisa594, PE or APC were added and incubated for one hour at room temperature. Nuclei were counterstained with 4′,6-diamidino-2-phenylindole (DAPI). Stained mammospheres and monolayer cultured cells were imaged on a Confocal Microscope and Single Molecule detection system (Leica, TCS-SP5-MP-SMD).

### Cell sorting and analysis by flow cytometry

Cell sorting and analysis by flow cytometry were performed as described previously [14]. Briefly, 1 × 10^5^ single suspension cells prepared from xenograft tumor were incubated with a specific antibody on ice for 30 minutes. The mouse cells were stained with anti-H2Kd (BD Pharmingen™, 1:200) followed by PECy7-labeled secondary antibody (Jackson Labs, 1:250) on ice for 20 minutes. 7AAD-perCP5.5 (BD Biosciences, 1:100) was used to exclude the dead cells. An ALDEFLUOR assay kit (StemCell Technologies) was used to identify the cells with high ALDH activity as previously reported [15], which was treated with specific ALDH inhibitor, diethylaminobenzaldehyde (DEAB). BD FACS AriaTMIIU flow cytometer (Becton Dickinson) was used to sort the cells and FACSCanto (Becton Dickinson) was used to analysis the expression of indicated markers.

### Mammosphere assay

To evaluate the potential of mammosphere formation from sorted cells, a density of 1 × 10^3^ cells/ml was plated in an ultra-low attachment 24-well plate (Corning, Acton, MA, USA) and cultured in Ham’s F-12 serum-free medium (BioWhittaker) supplemented with BSA (0.4%), B27 (Invitrogen, Carlsbad, CA, USA), basic epidermal growth factor (bEGF) (20 ng/ml), hydrocortisone (1 μM, Sigma) and epidermal growth factor (20 ng/ml, BD Biosciences, CA, USA). Twelve days after culture, the numbers of mammospheres were counted using an inverted microscope.

### Differentiation of human tumor stem cells

Single suspension ALDH^+^ or ALDH^+^/GD2^+^ (3 × 10^5^) cells isolated from PTs (BC-P007, BC-P107 and BC-P515) were induced to differentiate into adipocytes with reagents, including dexamethasone (1 μM, Sigma), insulin (5 μg/ml, Sigma), isobutylmethylxanthine (0.5 mM, Sigma), and indomethacin (60 μM, Sigma). The culture medium was refreshed once a week for 30 days. For the differentiation of osteocytes, cells were incubated with ascorbic acid (50 μg/ml, Sigma), β-glycerophosphate (10 mM, Sigma), and dexamethasone (10^-7^ M, Sigma), and observed after 20 days. For the differentiation of chondrocytes, 1 × 10^5^ cells were cultured in Dulbecco’s modified Eagle’s medium supplemented with Insulin-Transferrin-Selenium (ITS,50 mg/ml, GIBCO, USA), sodium pyruvate (1 mM, GIBCO), TGF-β (10 ng/ml, Pepro Tech, Inc.) and dexamethasone (10^-7^ M, Sigma), and the chondrocytes were observed at Day 15. For neural stem cell, ALDH^+^/GD2^+^ (5 × 10^3^) cells were cultured in Dulbecco’s modified Eagle’s medium supplemented with basic fibroblast growth factor (bFGF) (5 ng/ml, Pepro Tech, USA), retinoic acid (0.5 μM, Sigma) and 2-mercaptoethanol (1 mM) at Day 1; cyclic adenosine monophosphate (cAMP) (1 nM, Sigma) and ascorbic acid (100 μM, Sigma) at Day 3; cAMP (1 nM, Sigma) and hydrocortisone (10 μM, Sigma) at Day 5; nerve growth factor (NGF) (10 ng/ml, Sigma), epidermal growth factor (EGF) (1 mM, BD), butylated hydroxyanisole (200 μM, Sigma) and ITS + premix (50 mg/ml, GIBCO) at Day 8.

### Special stains

The differentiated adipocytes were confirmed by Oil Red O staining as reported. Briefly, cells were fixed with 4% paraformaldehyde and then stained with Oil Red O (0.3%, Sigma) for 15 minutes at room temperature. Osteoblasts were confirmed by staining cells with Alizarin Red S (0.5%, Sigma) for 10 minutes at room temperature. For chondroblasts, Alcian blue staining was performed. Cells were stained with an Alcian blue (pH = 2.5) kit (MUTO, Japan) for approximately 15 to 25 minutes at room temperature. After washing with acetic acid (3%), Kernechtrot solution was used for a counter stain.

## Results

### Successful engraftment of primary human PTs in NOD-SCID mice

**Figure 1:**
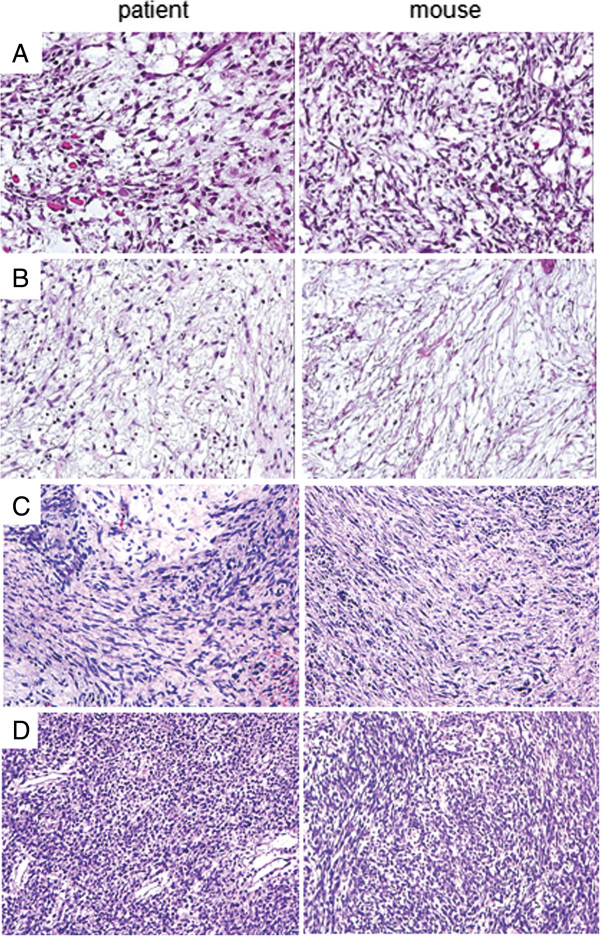
**Histopathology of human tumor and xenografted tumors.** Histopathology of patient primary tumors, BC007 **(A)**, BC107 **(B)**, BC515 **(C)** and BC877 **(D)** and their engrafted tumors (BC-P007MT2, BC-P107MT1, BC-P515 MT1 and BC-P877 MT1) were examined by H&E staining 200×.

Using strategies developed by Dialynas *et al*. [16] and Yu (unpublished), fresh surgical specimens obtained from four patients with malignant PTs were successfully engrafted into NOD-SCID mice as illustrated in Figures 1A-D. For primary BC007 PT cells, the initial engrafted tumor was harvested on Day 184 after inoculation and serially propagated in NOD-SCID mice for up to nine generations. It was noted that the growth rate of the tumors accelerated with each passage, reaching 1 cm in diameter within 40 days at the ninth generation. Similarly, primary BC107, BC515 and BC877 PT cells were xenografted in NOD-SCID mice and harvested around Day 200. The histological features of four engrafted tumors, designated as BC-P007, BC-P107, BC-P515 and BC-P877 as examined by H&E staining were very similar to their primary counterparts (Figure 1).

### Phenotypic markers of primary PTs and xenografts

Paraffin blocks from 47 individual patients, four fresh specimens of malignant PT and their xenografted tumors, were examined by immunohistochemical staining for the following markers: CD44 (HCAM), CD29 (integrin β-1), CD106 (VCAM-1), CD166 (ALCAM), CD105 (Endoglin), CD90 (Thy-1) [17-20], GD2 (ganglioside) [11], CD117(c-kit receptor) [21], ALDH1 [15], embryonic stem cell marker Oct-4 (Octamer-4 in abbreviation), CD34 [22], CD10, p53, p63, Ki-67, Bcl-2 [23] and vimentin [24]. Mesenchymal progenitor cell-line HS-5 was chosen as a control. We found that all markers were detectable in the 51 malignant PT specimens with the following frequencies among different patients: GD2 (100%, 51/51) CD166 (78.4%, 40/51), CD90 (78.4%, 40/51), CD44 (68.6%, 35/51), CD106 (29.4%, 15/51), CD29 (27.4%, 14/51) and CD105 (1.9%, 1/51) (Table 1). Vimentin was expressed in all tumor specimens, consistent with their mesenchymal lineage [25]. CD117 was expressed in 70.5% of the malignant PT specimens which is in line with the report of its expression in 13% and 67%, of benign and malignant PTs, respectively [26]. Ki-67, a marker for cell proliferation, was expressed in 82.3% of the malignant PT specimens. CD10 was detected in 60.7% (positive: ≥10%) [4] of the malignant PT specimens, which is consistent with the previous report of its presence in four of six malignant PTs but negative in all benign ones [4]. Overexpression of p53 was noted in 50.9% of the malignant PT specimens, including four fresh tumors as well as their xenografted tumors. Interestingly, the expression of p63, a member of the *p53* gene family highly expressed in the basal or progenitor layers of many epithelial tissues, was observed in 9.8% of the malignant PT specimens. Bcl-2 expression was found in 37.2% of the malignant PT specimens, but not in the four fresh primary tumors and their xenografted tumors. CD34, a transmembrane glycoprotein expressed on hematopoietic stem and progenitor cells, endothelial cells, bone marrow progenitor cells, and many mesenchymal tumor cells [27], was detected in 52.9% of the malignant PT specimens. Globo H, a hexasaccharide antigen commonly found in breast carcinoma (61 to 80%) [14,28], was noted in 9.8% of the malignant PT specimens. As summarized in Table 1, results from the immunohistochemical analysis showed that malignant PTs possess MSC-like properties and that the four fresh malignant PT samples and their corresponding xenografts showed largely similar immunohistochemical profiles as their parent tumors, up to the eighth passage (Additional file 1: Table S1). Consistent with their origin from stromal cells, these four primary malignant PTs, their non-tumor part, and their xenografts all lacked cytokeratins, but expressed vimentin except non-tumor parts of patient BC515 (Additional file 1: Table S2). In addition, we examined the phenotypes of non-tumor part (515NT and 877NT) by immunohistochemical analysis and showed that their phenotypes were mostly different from their original tumors and xenografts (Additional file 1: Table S1).

**Table 1:** Expression of various markers in PTs obtained from patients or patient-derived xenografts

		**% positive cases (n = 51)**	**BC007/BC-P007MT1**	**BC107/BC-P107MT1**	**BC515/BC-P515MT1**	**BC877/BC-P877MT1**	**HS-5**
		**(% positive cells)**					
MSC marker	CD44	68.6%	50/60	60/30	-/-	-/-	+
	CD29	27.4%	10/40	<5/30	5/<1	-/-	+
	CD166	78.4%	<5/< 5	10/50	5/5	90/90	+
	CDI06	29.4%	30/<10	<10/<10	-/-	80/80	+
	CD105	1.9%	-/-	-/-	-/-	50/90	+
	CD90	78.4%	<10/< 10	10/10	55/75	<5/70	+
	CD117	70.5%	-/20	-/-	5/50	80/95	+
	GD2	100%	80/50	75/30	90/75	60/30	+
		(<10 to >90)					
Stem cell marker	ALDH	100%	20/1	3-5/<1	40/5	<1/<5	+
		(<10 to >90)					
	Oct-4	47.0%	10/5	30/10	<1/35	<5/<5	+
PT clinically relevant markers	Vimentin	100%	90/ > 90	90/>70	80/20	>95/>95	+
		(<10- to >90)					
	Ki-67	82.3%	40/40	10/20	5/<3	65/>95	+
	CD10	60.7%	20/5	10/70	5/5	<10/30	+
	CD34	52.9%	-/-	40/70	80/20	-/-	-
	p53	50.9%	<10/<10	<5/10	70/60	>95/>95	+
	Bcl-2	37.2%	-/-	-/-	-/-	-/-	-
	p63	9.8%	60/10	-/-	-/-	-/-	-
	Globo-H	9.8%	-/-	-/-	-/-	80/80	-

Fifty-one malignant PTs were tested by immunohistochemical staining for a panel of 18 markers. The expression level of each marker was scored based on the positive percentage by stained cells by clinical pathologists. The percentage of all 51 cases expressing each marker is listed in the third row. The xenografted tumors in NO-SCID mice (BC-P007MT1, BC-P107 MT1, BC-P515 MT1 and BC-P877 MT1) showed a similar immunohistochemical staining profile for most markers as did the parental tumors (BC007, BC107, BC515 and BC877). All markers were expressed in malignant PTs. HS-5 (human marrow stromal cell lines) as the control in this table, the expression level of each marker was classified into two categories, negative (-) and positive (+), by clinical pathologists.

### ALDH^+^ identifies cancer stem cells in malignant PTs

**Figure 2:**
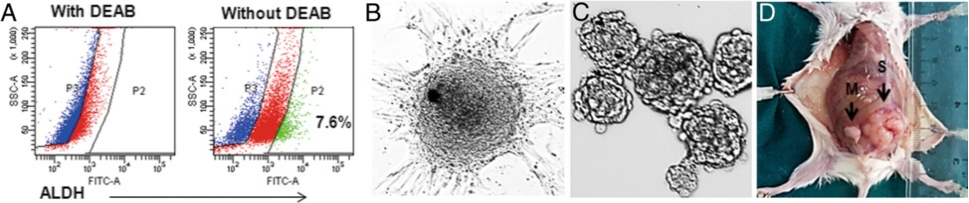
**Features of ALDH**^**+ **^**cell population in monolayer and mammosphere culture. (A)** Flow cytometry revealed 7.6% of the xenografted tumor cells in BC-P007MT3 (passage 3) were positive for ALDEFLUOR assay. **(B)** The ALDEFLUOR-positive cells were incubated in a culture dish, and formed colonies spontaneously (200×). **(C)** BC-P007 ALDH^+^ cell are capable of generating mammospheres in culture (200×). **(D)** As few as 50 ALDH^+^ harvested from mammospheres (S) or monolayer (M) were sufficient for tumor engraftment. ALDH, Aldehyde dehydrogenase 1.

ALDH has been shown to enrich breast stem/progenitor cells [29]. We thus examined the expression of ALDH in malignant PTs. As shown in Figure 2A, a small fraction (7.6%) of the xenografted tumor cells from BC-P007 was found to be positive for ALDH activities as determined by an ALDEFLUOR assay. The percent of cells with high ALDH activity in four xenografted tumors ranged from 3 to 30%. Several lines of evidence indicated that ALDH^+^ cells displayed features of tumor stem cells. First, the sorted ALDH^+^ cells spontaneously formed colonies adherent to the monolayer culture dish (Figure 2B). This phenomenon was observed in the monolayer cultures derived from all four xenografts. The colony forming efficiencies for BC-P007 and BC-P515 were approximately 14.1 to 16.6/10^6^ and approximately 15.5 to 17.5/10^6^, respectively, while those for ALDH^-^ cells were only approximately 0.8 to 1.6/10^6^ and approximately 0.11 to 0.23/10^6^, respectively (Additional file 1: Table S3). Upon trypsinization and replating, the colony formation persisted through serial passages for 20 passages, but the number of colonies declined gradually with each passage, along with a reduced growth rate. On the other hand, the ALDH^-^ cell population lasted for one or two passages in monolayer culture only with occasional colony formation.

**Figure 3:**
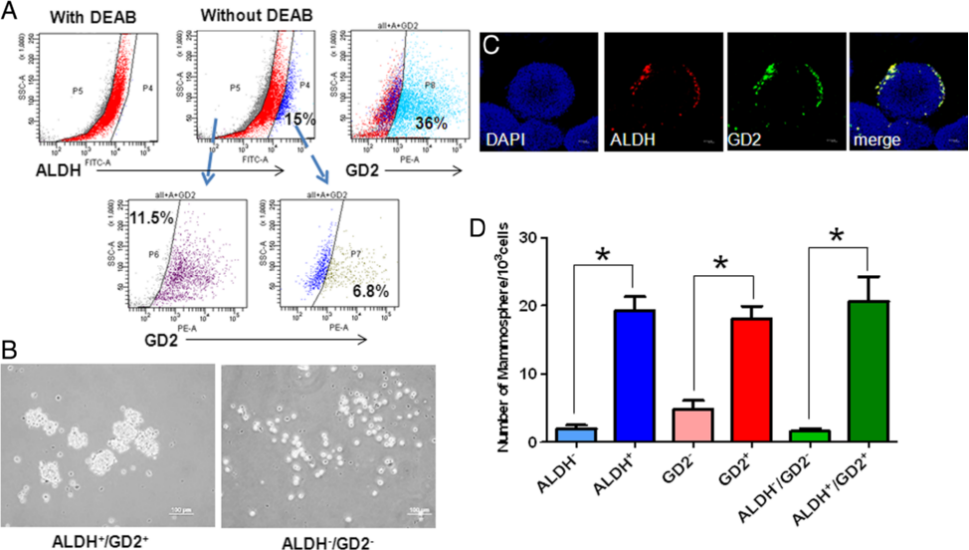
**Characteristics of ALDH**^**+**^**/GD2**^**+ **^**cells. (A)** The expression of Aldehyde dehydrogenase 1 (ALDH) and GD2 on xenografted BC-P007 cells was determined by flow cytometry. **(B)** ALDH^+^/GD2^+^ and ALDH^-^/GD2^-^ cells were sorted from the xenografted BC-P007 cells and cultured in a mammosphere condition at 1,000 cells/well of 24-well plates to mammosphere formation. Representative images of mammospheres formed from ALDH^+^/GD2^+^ (left panel) and ALDH^-^/GD2^-^ (right panel) cells were shown. The distribution of ALDH and GD2 expression on the mammospheres was examined by immunofluorescent microscopy with ALDH-AF594/GD2-AF488 antibodies. Single confocal sections **(C)** of mammospheres stained for ALDH (red), GD2 (green) and nucleus (blue) are presented. **(D)** The mammosphere forming capacity of the indicated cell populations sorted from BC-P007, BC-P107 and BC-P515. The mammospheres of the indicated cell populations of these three xenografted tumor cells were counted and their mean mammosphere formation efficiency was presented as the mean ± SEM of triplicate experiments from each of the three independent xenografts. *, *P* <0.0001.

Secondly, ALDH^+^ cells sorted from BC-P007, BC-P107 and BCP-515 were able to generate mammospheres which could be propagated for at least 10 passages (Figure 2C). The average mammosphere forming efficiency (MFE) of ALDH^+^ cells from BC-P007, BC-P107 and BC-P515 was 19 ± 2.0/1,000 cells as compared to 1.9 ± 0.5/1,000 cells for ALDH^-^ cells (*P* <0.0001) (Figure 3D). Using limiting dilution of ALDH^+^ BC-P515 cells at one cell/well, we observed that a single cell could give rise to mammosphere formation, supporting its clonal origin (Additional file 1: Table S4). Curiously, MFE was much higher in the single cell experiments (approximately 7.1 to 11.8%) than in the bulk experiments (2.8%). This suggested that clumping of cells when seeded in bulk might have accounted for their lower MFE. Immunofluorescence analysis of colonies in monolayer culture or mammopheres revealed high expression of the following markers: CD29, CD44 and CD166. Interestingly, CD44 appeared to concentrate at the colony periphery, while CD10, CD29 and CD166 localized in the center of the colony (Additional file 1: Figure S1A-C and E-G). ALDH (antibody) positive cells also congregated at the colony or mammosphere periphery (Additional file 1: Figure S1D-H).

Lastly, the *in vivo* tumorigenicity of ALDH^+^ and ALDH^-^ cells sorted from BC-P007 and BC-P515 xenograft were examined in NOD-SCID mice. The tumor formation frequency for ALDH^+^ cells (1 in 2 × 10^4^) from both xenografts was estimated to be 5- and 21-fold that of ALDH^-^ cells ( 1 in 1.1 × 10^5^ and 1 in >4.2 × 10^5^) for BC-P007 and BC-P515, respectively, as shown in Table 2. Interestingly, ALDH^+^ cells from monolayer cultures and mammospheres derived from BC-P007 xenografts were even more tumorigenic than ALDH^+^ cells freshly sorted from xenografts (Additional file 1: Table S5). As few as 50 ALDH^+^ cells from mammospheres or monolayer cultures were sufficient for tumor engraftment (Figure 2D). The frequencies of tumor formation for cultured ALDH^+^ cells (1 in 53 for monolayers, 1 in 1 for mammospheres) were significantly higher than that of ALDH^+^ cells sorted from BC-007 xenografts without culture (*P* <0.001; *P* <0.001, respectively; Additional file 1: Table S5) but there was no significant difference between the monolayer- and mammosphere-derived ALDH^+^ cells (*P* = 0.3). On the other hand, the tumor formation frequency of ALDH^-^ cells derived from monolayers were significantly lower than that of ALDH + cells (1 in 1.8 × 10^5^ vs. 1 in 53, *P* <0.001; Additional file 1: Table S5). Collectively, these findings suggest that ALDH^+^ cells sorted from BC-P007 and BC-P515 xenografts, were enriched in PT stem cells, which were further enriched by *in vitro* cultures as monolayers or mammospheres.

**Table 2:** Engraftment capacity of different cell populations sorted from cryopreserved tumor cells

**BC-P007**	**4 × 10**^**4**^	**2 × 10**^**4**^	**1 × 10**^**4**^	**5 × 10**^**3**^	**4 × 10**^**3**^	**1 × 10**^**3**^	**500**	**100**	**50**	**20**	**Frequency**^**〒**^	**Distribution**^**#**^
ALDH^+^		3/8			3/8	1/3	1/7				1:2.0 × 10^4^	75%
ALDH^-^		0/4			0/6	1/7	0/3	0/4			1:1.1 × 10^5^	25%
ALDH^+^/GD2^+^		2/2			3/3	6/10		5/10	2/20	0/3	1:6.1 × 10^2^	
ALDH^-^/GD2^-^	0/4		0/1	0/8	2/10	0/15					1:1.3 × 10^5^	
ALDH^+^		3/8			3/8	1/3	1/7				1:2.0 × 10^4^	
ALDH^+^/GD2^+^		2/2			3/3	6/10		5/10	2/20	0/3	1:6.1 × 10^2^	
**BC-P515**	**1 × 10**^**5**^	**2 × 10**^**4**^	**1 × 10**^**4**^		**4 × 10**^**3**^	**1 × 10**^**3**^	**500**	**100**		**50**	**Frequency**^**〒**^	**Distribution**^**#**^
ALDH^+^		1/4	0/3		1/4	3/9		1/5			1:2.0 × 10^4^	96%
ALDH^-^		0/4	0/3		0/2	0/9					1:>4.2 × 10^5^	4%
ALDH^+^/GD2^+^		1/3*	7/10			4/5	0/1	2/7		1/2	1:7.1 × 10^3^	
ALDH^-^/GD2^-^	0/1	0/2	1/9		0/4	0/10					1:2.5 × 10^5^	
ALDH^+^		1/4	0/3		1/4	3/9		1/5			1:2.0 × 10^4^	
ALDH^+^/GD2^+^		1/3	7/10			4/5	0/1	2/7		1/2	1:7.1 × 10^3^	

BC-P007 and BC-P515 cryopreserved tumor cells from patient-derived xenografts of PT were sorted by FACS into ALDEFLUOR-positive, ALDEFLUOR-negative or ALDEFLUOR and GD2 double positive and double negative cells. Varying numbers of the sorted cells were injected in fat pads of NOD-SDID mice to evaluate their tumor-initiating potential. The frequency of cancer-initiating cells was analyzed using ELDA software.

*:Including one of three mice died the day after tumor injection.

#:The distribution of tumor-initiating cells among the BC-P007 and BC-P515 ALDH + and ALDH- cells.

〒:Frequency was calculated by ELDA software, although the *P*-value for likelihood ratio test of single-hit model in each group was less than *P* = 0.5.

### Comparison of primary cultures of cells derived from the tumor and non-tumor part of PT

To address the possibility that the mammmospheres may be derived from ALDH^+^ normal stromal cells, we established primary cultures of cells derived from the tumor part and non-tumor part of human breast cancer sample BC515. The primary culture of the non-tumor part (515-NT) could be propagated for up to approximately 18 to 19 passages only, whereas the tumor part can be sustained *in vitro* for at least 35 passages. The tumorigenic ability of 515NT was tested by injecting 2.5 × 10^6^ cells of 515 NT into the cleared fat pads of 12 mice. None of these mice showed tumor growth up to Day 291. On the other hand, injection of 1 × 10^6^ cells of BC-P515 yielded tumor growth in 20/20 mice by Day 7 after injection. In addition, the ALDH^+^ and ALDH^-^ cells of BC-P515 and 515NT were sorted to evaluate their ability for mammosphere formation. As shown in Additional file 1: Table S6, the number of mammospheres was at least one log higher (10-fold) in ALDH^+^ BC-P515 cells than in ALDH^+^ 515 NT cells. Furthermore, BC-P515 contained 40 XO chromosomes with several karyotypic abnomalities, in contrast to a normal karyotype with 46 XX in 515 NT (data not shown).

### ALDH^+^/GD2^+^ cells could serve as a marker for cancer stem cells in malignant PTs

To screen for other markers of cancer stem cells in PTs, we harvested the BC-P007 xenografted tumor cells for FACS sorting using a panel of markers including CD29, CD44, CD90, CD117, CD 133, CD166, GD2, CD10, CD24 and ALDH. Following cell sorting, various subpopulations of the BC-P007 xenografted tumor were evaluated for their ability to grow as monolayer cultures with serial passages. CD24 and CD133 were not expressed in BC-P007 and subpopulations sorted by CD10, CD90, CD117 and CD166 grew very poorly in monolayer cultures. CD29^+^ and CD44^+^ cells lasted for two to three passages only. The neural ganglioside GD2 was reported to be a marker for MSC and GD2-positive MSC could be induced to differentiate into osteoblasts, adipocytes and chondroblasts [10]. In addition, GD2 expression was observed in 25% to 67% of the cells from four xenografted tumors. Thus, GD2 was also included in the panel for testing. Notably, GD2^+^ cells propagated for more than 18 generations whereas GD2^-^ cells did not last for more than three passages. Furthermore, ALDH^+^/GD2^+^ (6.8%) and ALDH^-^/GD2^-^cells (11.5%) were sorted from BC-P007 xenograft (Figure 3A), and cultured in mammosphere condition. As shown in Figure 3B, ALDH^+^/GD2^+^ cells formed significantly more mammospheres than double negative cells. Immunofluorescence staining showed the preferential localization of ALDH^+^/GD2^+^cells at the sphere periphery (Figure 3C, Z-stack). Next, we compared the mammosphere forming capacities for each subpopulation of BC-P007, BC-P107 and BC-P515 xenografts based on their ALDH and GD2 expression. As shown in Figure 3D, MFE of GD2 positive cells from these three xenografts was 18.08 ± 1.7/1,000 cells, as compared to 4.8 ± 1.2 for GD2 negative cells (*P* <0.0001), and MFE of ALDH^+^/GD2^+^ cells was 20.5 ± 3.6/1,000 as compared to 1.6 ± 0.4 for ALDH^-^/GD2^-^ cells (*P* <0.0001). These results suggested that ALDH/GD2 single and double positive cells might have greater tumorigenic potentials than single and double negative cells. As a further proof, cryopreserved BC-P007 and BC-P515 xenografted cells were sorted into single or double positive and double negative cells for ALDH and GD2 and injected into NOD-SCID mice. Significantly, the tumor-initiating frequency of ALDH^+^/GD2^+^ cells sorted from cryopreserved BC-P007 and BC-P515 was estimated to be approximately 35.2- to 1,000- and approximately 2.8- to 33.3-fold higher than ALDH^-^/GD2^-^ and ALDH^+^ cells, respectively (Table 2). In addition, the phenotypic profile of xenograted tumors derived from ALDH^+^/GD2^+^ cells showed intratumor heterogeneity, similar to the parental tumors, and the sorted ALDH^+^/GD2^+^ cells could be serially passaged in mice for more than three generations (Additional file 1: Table S7).These findings support the notion that ALDH^+^/GD2^+^ expression could serve as a marker to enrich cancer initiating cells in malignant PTs.

### *In vitro* differentiation of ALDH^+^/GD2^+^ cells into neuro-ectodermal cell lineages

**Figure 4:**
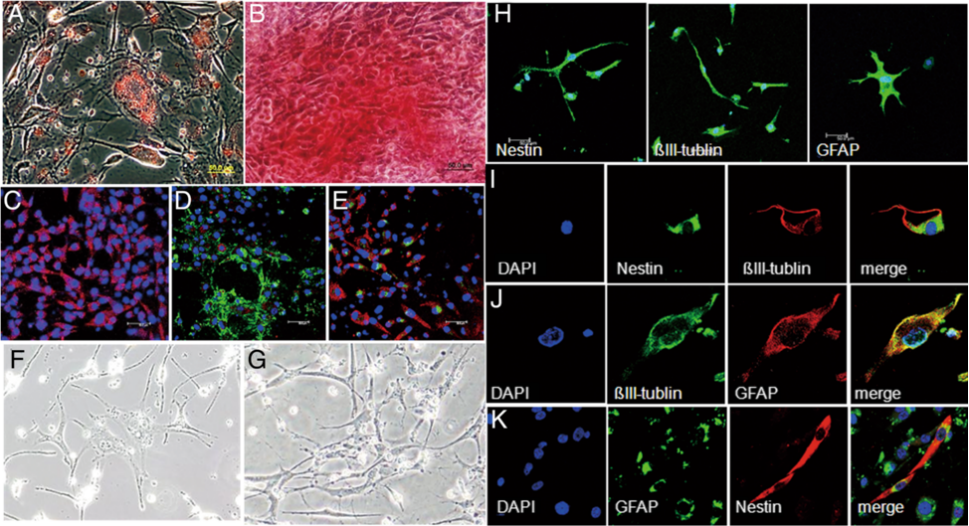
***In vitro *differentiation of ALDH**^**+**^**/GD2**^**+ **^**cells into neuro-ectodermal lineages.** Monolayer cultures of Aldehyde dehydrogenase 1 (ALDH)^+^ cells obtained from BC-P007 xenografts were induced to differentiate into various cells lineages. Adipocyte was induced by incubation with dexamethasone, insulin, isobutylmethylxanthine and indomethacin for 30 days, and identified by Oil Red O staining (**A**. 400×). Osteocytes were induced by treatment with ascorbic acid, β-glycerophosphate and dexamethasone for 23 days and examined by Alizarin red staining (**B**. 400×). Chondrocytes were induced by culturing with ITS + premix, sodium pyruvate, TGF-β and dexamethasone for 15 days, and confirmed by immunofluorescence staining with anti-human collagen II antibody (**C**. 400×). The expression of GD2 (green) and collagen type 2 (red) in chondrocytes was detected by fluorescence-conjugated antibodies and observed under fluorescence microscope (**D** and **E**. 400×). Neuron-like cells were induced by incubation of the ALDH^+^/GD2^+^ cells from xenografted tumor of BC-P007 with retinoic acid, hydrocortisone, ITS and c-AMP, and was observed under phase contrast microscope on Day 7 (**F**. 400×) and Day 14 (**G**. 400×). Twenty-two days after culture, the neuronal lineages of these cells were examined by fluorescence microscopy with AF488 labeled antibodies against nestin, βIII-tubulin and GFAP (**H**. 400×). And, double staining of nestin and βIII-tubulin, βIII-tubulin and GFAP and GFAP and nestin (**I** - **K**, 1,000×) with AF488 or AF594-labeled antibodies was shown.

We next evaluated the potentials of ALDH/GD2 single positive and double positive cells from BC-P007, BC-P107 and BC-P515 xenografted tumors to differentiate into various cell lineages. Using an *in vitro* culture system, we found that the ALDH^+^ cells sorted from BC-P007 xenografted tumors could be induced to differentiate into adipocyte as revealed by Oil Red O staining (Figure 4A), osteocytes with positive staining by Alizarin red (Figure 4B), and chondrocytes, with immunofluorescent staining for collagen type II (Figure 4C). Furthermore, GD2 and collagen type II were expressed by different populations of cells in cultures induced for chondrocyte differentiation (Figure 4D, E). When ALDH^+^/GD2^+^ cells were induced for neuronal differentiation with retinoic acid, hydrocortisone, ITS and cAMP, spindle-shaped cells emerged on Day 7 (Figure 4F), which displayed a network architecture by Day 14 (Figure 4G). By Day 22, these cells differentiated into different neural lineages, as identified by specific neural markers, including nestin (neuron stem/progenitors cell marker), βIII-tubulin (immature neuronal progenitor cell marker) and GFAP (glial fibrillary acidic protein, marker for astrocytes) (Figure 4H). Interestingly, cells staining for nestin in the cell body and βIII-tubulin in the tail portion were noted occasionally (Figure 4I). In addition, a few cells displayed co-localized staining for GFAP and βIII-tubulin (Figure 4J). On the other hand, nestin and GFAP expression was observed in separate cell populations (Figure 4K). These findings demonstrated the ability of ALDH^+^/GD2^+^ cells to differentiate into diverse lineages of neural cells. Furthermore, the ALDH^+^/GD2^+^ cells could also be induced to differentiate into adipocytes, osteocytes and chondrocytes, comparable to ALDH^+^ cells. Similar results were observed with ALDH^+^ or ALDH^+^/GD2^+^ cells sorted from the BC-P107 and BC-P515 xenografted tumors (data not shown). Our findings suggest that ALDH^+^/GD2^+^ possess the ability to differentiate along various cell lineages, including neuro-ectodermal lineages. This is the first documentation for neural differentiation of malignant PT *in vitro*.

### Capacity of ALDH^+^ or ALDH^+^/GD2^+^ cells to undergo spontaneous differentiation *in vivo*

**Figure 5:**
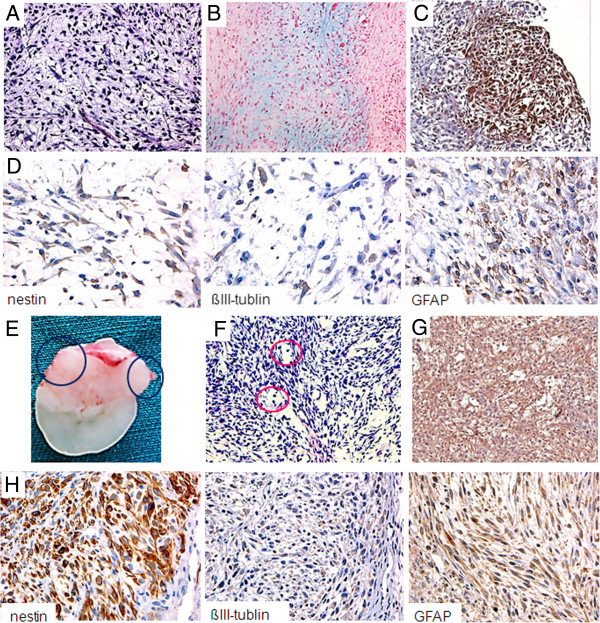
**Capacity of ALDH**^**+ **^**and ALDH**^**+**^**/GD2**^**+ **^**cells to undergo differentiation *in vivo*.** Mammospheres derived from BC-P007 Aldehyde dehydrogenase 1 (ALDH)^+^ cells were engrafted. Five thousand ALDH^+^ cells were injection into fat pads and on Day 86, severe tumor nodules were observed in the thoracic cavity. In addition to tumor at the injection site, one of the metastatic tumors removed for H&E staining revealed the presence of cells with lacunar space, suggestive of chondroid cells (**A**. 200×), which was confirmed by Alcian blue staining (**B**. 200× ) and anti-collagen type II staining (**C**. 200×). In addition, the metastatic tumor was examined by staining for neuro-ectodermal cell lineages markers, including nestin, ßIII-tublin and GFAP (**D**. 400X). Fifty ALDH^+^/GD2^+^ cells sorted from the BC-P007 xenografted tumor were engrafted and the tumor was removed on Day 61**(E)**. H&E staining revealed the presence of neuron-like cells (circles) (**F**. 200×). The tumor was examined for the expression of collagen type II, nestin, βIII-tublin and GFAP by immunohistochemical staining. The right upper region expressed anti-human collagen type II (**G**. 200×), and the left upper region expressed nestin moderate staining, βIII-tublin and GFAP expressed weak staining (**H**. 400×).

Among more than 16 BC-P007 xenografted mice, one-fifth showed metastatic lesions, mostly in the thoracic cavity. Some of the metastatic tumors contained cells with lacunar space easily discernible by H&E staining, reminiscent of chondroid cells (Figure 5A), which were confirmed by Alcian blue staining (Figure 5B) and immunohistochemical staining with anti-human collagen type II antibody (Figure 5C). Other lesions differentiated into different lineages of neural cells with the expression of nestin, βIII-tubulin and GFAP by immunohistochemical staining (Figure 5D). Interestingly, the primary tumor of BC-P007 xenograft at the site of injection expressed collagen type II (50% moderate staining, data not shown), but not nestin, βIII-tublin and GFAP. These findings suggested that mammospheres derived from ALDH^+^ cells possessed the ability to differentiate into various cell lineages *in vivo*. Similarly, mammospheres derived from ALDH^+^/GD2^+^ cells could form xenograft tumors with prominent features of neural and chondroid differentiation. Figure 5E showed an interesting tumor engrafted from ALDH^+^/GD2^+^ cells with a distinct dichotomy in differentiation. The right portion of the tumor stained positively for collagen type II (Figure 5F) and the left portion contained neuron-like cells by H&E staining (Figure 5G) with weak to moderate staining for nestin (70%), βIII-tublin (10%) and GFAP (60%, Figure 5H). In addition, clinical tumor specimens of patients BC007, BC107, BC515 and BC877 were also found to express nestin (approximately 20 to 80%), βIII-tublin (approximately <10 to 35%) and approximately GFAP (35 to 80%) (Additional file 1: Table S8). Thus, this *in vivo* system lends further support that ALDH^+^ and ALDH^+^/GD2^+^ subpopulations of malignant PTs might be enriched in cells displaying characteristics of MSC, namely the ability for self-renewal and differentiation into a variety of cell lineages including neural specification. To date, this is the first time that malignant PTs were shown to differentiate into neural lineage *in vivo.*

## Discussion

PTs were derived from the periductal stromal cells of the breast. Malignant stromal transformation in PT is usually of the fibrosarcomatous type [6,7]. Thus, PT lacks cytokeratins which are found in most carcinomas, carcinoid tumors and epithelial organs, but expresses vimentin which is found in tumors of mesenchymal origin, not carcinomas [24,30]. In line with this, for all PT samples in our study, their xenografted tumors displayed positive staining for vimentin and negative for cytokeratins. In addition to the common malignant fibrous elements, features of rhabdomyosarcoma, liposarcoma and osteosarcoma were occasionally observed in malignant PTs. The heterogeneity of malignant PTs has been largely attributed to metaplastic changes of the malignant stromal cells. The versatile properties of malignant PTs suggest that CSCs exist in PT. We demonstrated for the first time that many markers, including CD44, CD29, CD106, CD166, CD90, CD117 and GD2 were expressed in malignant PTs although CD105 was detected in only 1 of 51 PTs. CD105, a type I membrane glycoprotein that is found in endothelial cells, activated macrophages, fibroblasts and smooth muscle cells is a specific and sensitive marker for tumor angiogenesis. Its expression was associated with increased micro-vessel staining and poor prognosis in childhood’s acute lymphoblastic leukemia [31], breast carcinoma [32], colorectal carcinoma [33] and so on. However, whether CD105 is expressed in sarcoma or malignant PT has not been reported until now. Interestingly, Globo H, a carbohydrate antigen commonly expressed in breast cancer (61 to 80%), was also detected in malignant PT specimens, albeit at lower frequency (9.8%). Moreover, several groups have shown an association of the degree of malignancy of PTs with the expressions of Ki-67, CD117, CD10 and p53. Consistent with these reports, high frequency of malignant PTs expressing Ki-67 (82.3%), CD117 (70.5%), CD10 (60.7%) and P53 (50.9%) was observed in the present study. Furthermore, expression of CD117 (c-kit) was found to correlate significantly with both grades and recurrence of PTs [34]. Since c-kit-overexpressing cancers, including gastrointestinal stromal tumors, could be treated with tyrosine kinase inhibitor, such as imatinib mesylate, it will be of interest to investigate whether malignant PTs may be responsive to c-kit inhibitors.

CSCs play a significant role in the survival and progression of malignant neoplasms [35]. CD44^+^/CD24^-^ have been identified as cancer stem cell markers for breast cancer [36], CD133^+^ aimed at brain tumors [37] and colon cancer [38], as well as CD20 designed for melanoma [39]. More recently, CD45^-^/ CD90^+^, CD133^+^ and CD44 ^+^ have been identified as CSC markers for hepatoma [40] osteosarcoma [41] and stomach cancer [42], respectively. However, CSC markers for PT have remained an enigma. In this study, we demonstrated for the first time that ALDH^+^ cells could serve as a marker for enrichment of cancer stem cells in malignant PTs. Moreover, ALDH^+^/GD2^+^ cells could further enrich CSCs by 33-fold of ALDH^+^ cells based on *in vivo* tumorigenic potentials. In addition to self-renewal, CSCs harbors the capacity for differentiation into heterogeneous cell lineages. Indeed, tumors engrafted from ALDH^+^ or ALDH^+^/GD2^+^ cells showed evidence of *in vivo* differentiation into chondrocytes and neural cells. Furthermore, ALDH^+^/GD2^+^ cells could be induced to differentiate into adipocytes, osteocytes and chondrocytes as well as neural lineages *in vitro*. Our observation of neural differentiation of clinical specimens of PTs and their xenografts provided the first evidence for neural differentiation of malignant PTs. Taken together, these data support the notion that ALDH and GD2 may serve as markers for enrichment of CSCs for malignant PTs. It is noteworthy that GD2 has recently been reported to be a marker for CSCs in adenocarcinoma of the breast [11]. The findings of GD2 as a CSC marker for PT and breast cancer have important therapeutic implication, in light of the recent success of anti-GD2 in the treatment of high risk neuroblastoma [43].

## Conclusions

In this study, analysis of 51 clinical specimens of malignant PTs documented the expression of many markers expressed by mesenchymal stem cells, as well as ALDH and Oct-4. Using four xenografts established from primary human PTs, we demonstrated for the first time that ALDH and GD2 could serve as novel markers for malignant PTs, and could enrich CSCs of PTs. Moreover, we provided the first evidence that the sorted ALDH^+^/GD2^+^ cells could be induced to differentiate into neural cells of various lineages, in addition to adipocytes, osteocytes and chondrocytes. Evidence of neural differentiation was also observed in clinical specimens and xenografts of malignant PTs. Our findings revealed that malignant PTs possess many characteristics of MSC, and ALDH and GD2 could enrich PT stem cells.

## Abbreviations

ALDH: Aldehyde dehydrogenase 1; CSCs: Cancer stem cells; GFAP: Glial fibrillary acidic protein; ITS: Insulin-Transferrin-Selenium; MFE: Mammosphere forming efficiency; MSCs: Mesenchymal stem cells; NOD-SCID mice: Non-obese diabetic-severe combined immunodeficient mice; PTs: Phyllodes tumors; TGF-β: Transforming growth factor beta.

## Competing interests

The authors indicate no potential conflicts of interest.

## Authors’ contributions

JJL performed most of the experiments, analyzed the data and wrote the paper. JY drafted the manuscript and the experimental design. CSH, GSL, HCL and KTY collected the specimens, supervised the project and participated in discussions of the study design. JTH and RJL helped with data analysis and interpretation, as well as with manuscript writing. FPC conceived and coordinated the study. KTY and ALY contributed to study conception, experimental design and manuscript writing. All authors have approved the final manuscript for publication and agreed to be accountable for all aspects of the work.

## Supplementary Material

Additional file 1: Table S1 Expression of various markers in tumor and non-tumor parts of PTs obtained from patients and their xenografted tumors in mice by immunohistochemical analysis. **Table S2.** Expression of vimentin and cytokeratin in tumor and non-tumor parts of PTs obtained from patients and their xenografted tumors by immunohistochemical analysis. **Table S3.** Colony forming assays of BC-P007 and BC-P515. **Table S4.** A comparison of mammosphere formation efficiency (MEF) of ALDH^+^ and ALDH^-^ subpopulations. **Table S5.** Engraftment of tumors in NOD/SCID mice with different cell populations sorted from monolayer cultured cells, mammospheres and cryopreserved xenografted cells of BC-P007. **Table S6.** Comparison of the *in vitro*/*in vivo* growth properties and differentiation capacity of cells harvested from the tumor and non-tumor parts of malignant PT, BC-P515. **Table S7.** ALDH^+^/GD2^+^ cells could be serially passaged in mice. **Table S8.** Clinical tumor specimens of patients expressed nestin, ßIII-tublin and GFAP. **Figure S1.** Immunofluorescence analysis of BC-P007 colonies and mammosphere culture. Click here for file
