# Supplementary material for: Malignant phyllodes tumors display mesenchymal stem cell features and aldehyde dehydrogenase/disialoganglioside identify their tumor stem cells
Source: Breast Cancer Res. 2014 Mar 26;16(2):R29. doi: 10.1186/bcr3631 (PMC4053203; doi:10.1186/bcr3631)
Supplement: Additional file 1: Table S1 — Expression of various markers in tumor and non-tumor parts of PTs obtained from patients and their xenografted tumors in mice by immunohistochemical analysis. Table S2. Expression of vimentin and cytokeratin in tumor and non-tumor parts of PTs obtained from patients and their xenografted tumors by immunohistochemical analysis. Table S3. Colony forming assays of BC-P007 and BC-P515. Table S4. A comparison of mammosphere formation efficiency (MEF) of ALDH+ and ALDH- subpopulations. Table S5. Engraftment of tumors in NOD/SCID mice with different cell populations sorted from monolayer cultured cells, mammospheres and cryopreserved xenografted cells of BC-P007. Table S6. Comparison of the in vitro/in vivo growth properties and differentiation capacity of cells harvested from the tumor and non-tumor parts of malignant PT, BC-P515. Table S7. ALDH+/GD2+ cells could be serially passaged in mice. Table S8. Clinical tumor specimens of patients expressed nestin, ßIII-tublin and GFAP. Figure S1. Immunofluorescence analysis of BC-P007 colonies and mammosphere culture. [file bcr3631-S1.docx]

**Additional file 1**

**Table S1**


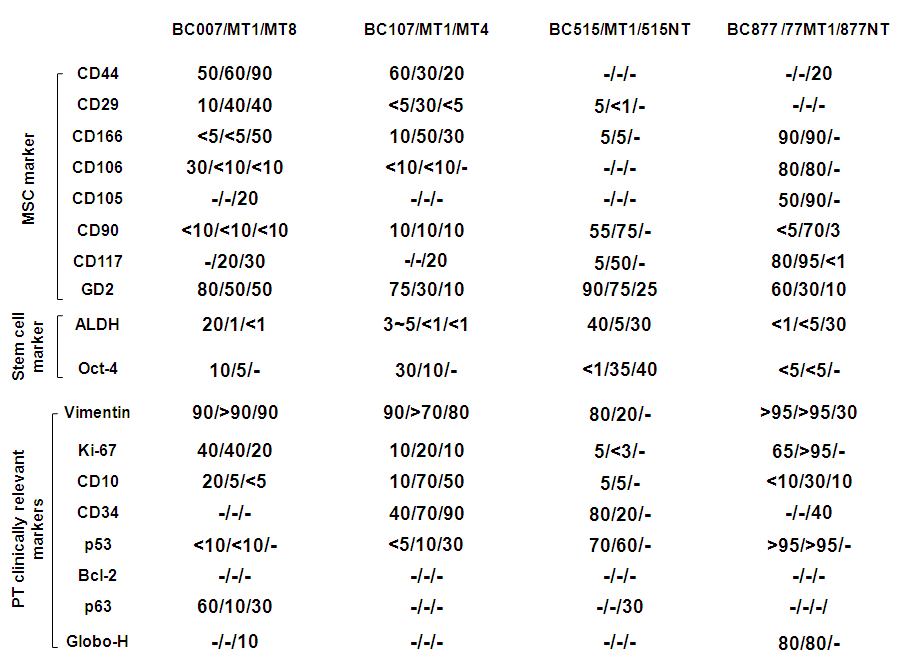


**Expression of various markers in tumor and non-tumor parts of PTs obtained from patients and their xenografted tumors in mice by immunohistochemical analysis.** Immunohistochemical staining for a panel of 18 markers was performed on the tumor and non-tumor parts (515NT and 877NT) of four malignant PTs samples and their serial passages of xenografted tumors (MT1 to MT8). The percentage of cells with positive staining was scored by clinical pathologists.

**Table S2**


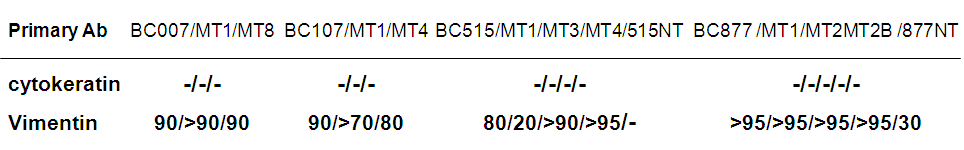


**Expression of vimentin and cytokeratin in tumor and non-tumor parts** of **PTs obtained from patients and their xenografted tumors by immunohistochemical analysis.** The tumor and non-tumor parts (515NTand 877NT) of four malignant PTs samples and their serial passages of xenografted tumors were tested by immunohistochemical staining for vimentin and cytokeratin. The percentage of cells with positive staining was scored by clinical pathologists.

**Table S3**


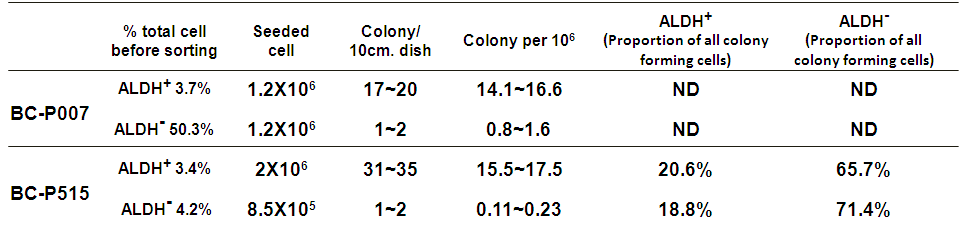


ND: not done

**Colony forming assays of BC-P007 and BC-P515.** ALDH^+^ and ALDH^-^ cells sorted from monolayer cultures of BC-P007 and BC-P515 were seeded on the 10cm Petri dishes at the indicated number of cells. The number of spontaneous colonies formed was counted at 7-10 days and the frequencies of colony-forming cells for both ALDH+ and ALDH- cells were determined. The proportion of all colony forming cells which were ALDH+ or ALDH- were shown in the last two columns.

**Table S4**


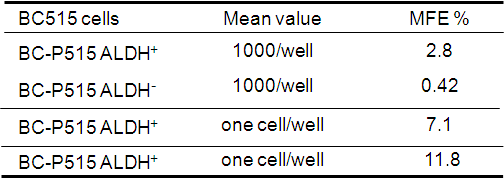


**A comparison of mammosphere formation efficiency (MEF) of ALDH+ and ALDH- subpopulations.** The mammosphere formation capacity of ALDH^+^ and ALDH^-^ of BC-P515 cells was determined at1000 cells/ well and one cell/well. The MFE was shown in the last columns.

**Table S5**


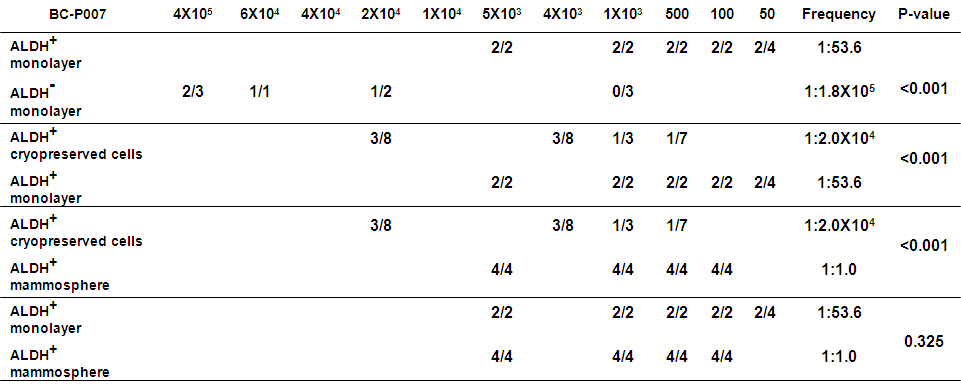


**Engraftment of tumors in NOD/SCID mice with different cell populations sorted from monolayer cultured cells, mammospheres and cryopreserved xenografted cells of BC-P007.** Monolayer cultured cells, mammospheres and cryopreserved derived from xenograft of primary human BC-P007 were sorted by FACS into ALDEFLUOR-positive, ALDEFLUOR-negative cells. Varying numbers of the sorted cells were injected in fat pads of NOD-SDID mice to evaluate their tumor-initiating potential. The frequency of cancer-initiating cells was analyzed using ELDA software.

**Table S6**


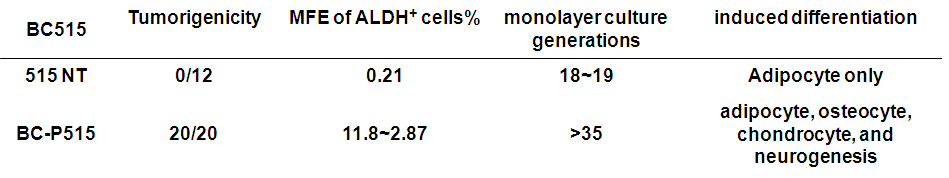


**Comparison of the in vitro/in vivo growth properties and differentiation capacity of cells harvested from the tumor and non-tumor parts of malignant PT, BC-P515.** Cells harvested from tumor part (BC-P515) and non-tumor part (515NT) of human breast cancer sample BC515 were placed in monolayer cultures for serial passages, or placed in mammosphere culture condition for determination of mammosphere forming efficiency (MFE). For in vivo tumorigenicity, 2.5X10^6^ cells of 515 NT or BC-P515 were injected into the cleared fat pads of NOD/SCID mice and observed for tumor formation up to day. To assess the differentiation capacity, BC-P515 and 515 NT cells were induced to differentiate into adipocytes, osteocytes, chondrocytes and neuron like cells, as described in Materials & Methods.

**Table S7**


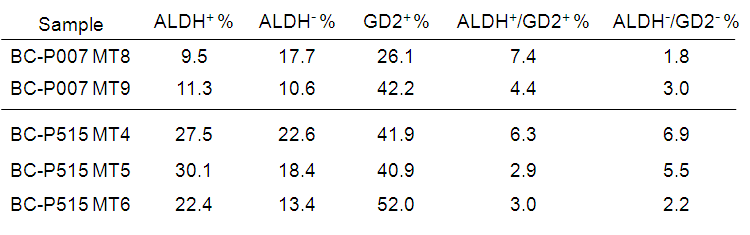


**ALDH^+^/GD2^+^ cells could be serially passaged in mice.** Serial passages of xenografted tumors derived from BC-P007 and BC-P515 ALDH^+^/GD2^+^ double positive cells were examined for the distribution of ALDH, GD2, and ALDH^+^/GD2^+^ double positive/negative subpopulations by FACS analysis

**Table S8**


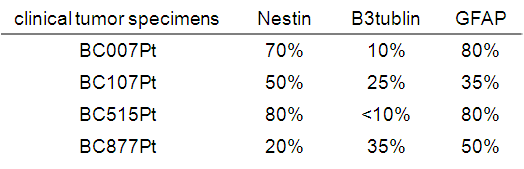


**Clinical tumor specimens of patients expressed nestin , ßIII-tublin and GFAP.** Four formalin-fixed and paraffin-embedded clinical malignant PTs were tested by immunohistochemical staining for nestin , ßIII-tublin and GFAP. The percentage of cells with positive staining was scored by clinical pathologists.

**Figure S1**


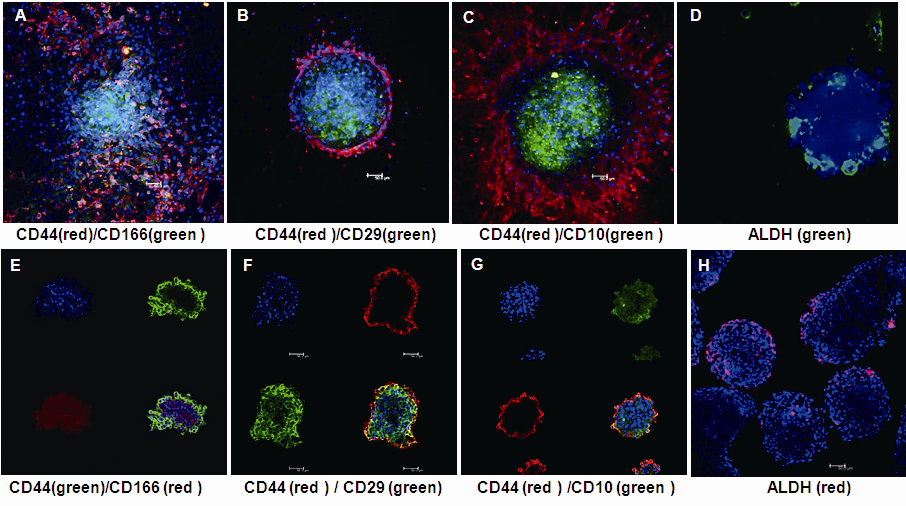


**Immunofluorescence analysis of BC-P007 colonies and mammosphere culture.** The spontaneous colonies formed in monolayer cultures of ALDH^+^ cells sorted from BC-P007 cells were stained with the following makers CD29, CD44, CD166, CD10 and ALDH (with anti-ALDH antibody), and the immunofluorescence was visualized under fluorescence microscopy (A~D). Immunofluorescent staining of mammospheres derived from BC-P007 ALDH^+^ cell of these same markers was examined (E~H).
